# Prereplication complex proteins get caught moonlighting

**DOI:** 10.1371/journal.pbio.3001549

**Published:** 2022-02-23

**Authors:** Hilary A. Coller

**Affiliations:** 1 Molecular, Cell and Developmental Biology, University of California, Los Angeles, Los Angeles, California, United States of America; 2 Department of Biological Chemistry, David Geffen School of Medicine, Los Angeles, California, United States of America; 3 Molecular Biology Institute, University of California, Los Angeles, California, United States of America

## Abstract

This Primer explores the implications of a PLOS Biology study that reports a novel function for proteins of the DNA pre-replication complex in promoting the anchor cell to invade through the basement membrane and initiate vulval development in Caenorhabditis elegans.

## Teaser

In this issue of *PLOS Biology*, Lattmann and colleagues report a new function for proteins of the DNA prereplication complex promoting the anchor cell to invade through the basement membrane and initiate vulval development in *Caenorhabditis elegans*.

Before the human genome was sequenced, guesses for the number of human genes ranged from over 300,000 to just under 26,000 [1]. Sequencing of the human genome revealed a surprise: There are only approximately 20,000 human genes. How could so few genes encode the information needed to create an organism as complex and sophisticated as us? Some of the answer certainly reflects the fact that 20,000 genes, through alternative splicing, can produce many more protein isoforms. Another part of the answer may be protein “moonlighting”—the ability of some proteins to perform 2 distinct functions, like individuals who work more than 1 job.

Examples of protein moonlighting exist in animals, plants, fungi, prokaryotes, and protists [2]. A significant fraction of the proteins in glycolysis and the tricarboxylic acid (TCA) cycle have a second, unrelated function [3]. Particularly surprising examples of moonlighting are the crystallin proteins that both ensure that eye lenses are transparent and exhibit catalytic activity as metabolic enzymes [3].

**Fig 1:**
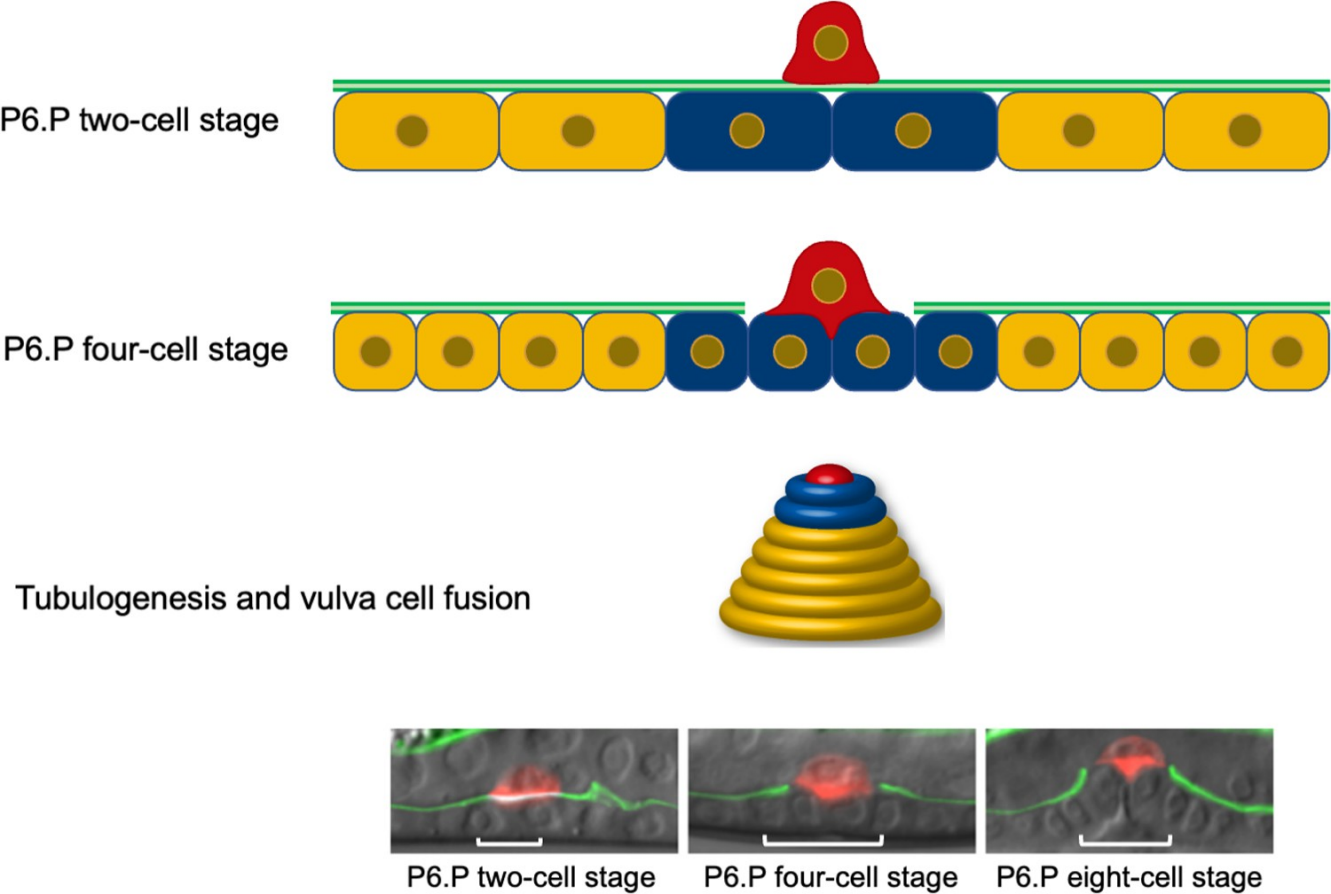
Schematic of *C*. *elegans* anchor cell invasion. The anchor cell must traverse the basement membrane to connect the uterus to the vulva. Lattmann and colleagues show that prereplication complex proteins are required for the ability of the anchor cell to invade through the basement membranes and induce the vulval progenitor cells to become vulva.

In this issue of *PLOS Biology*, Lattmann and colleagues report their discovery that a protein complex required for replicating DNA has an independent function in promoting invasion into the basement membrane [4]. The authors were investigating anchor cells in the roundworm *Caenorhabditis elegans*. During development, the anchor cell needs to pass through 2 basement membranes in order to connect the uterus to the vulva (Fig 1).

Using a genetic screen, the authors found that knocking down a gene involved in the prereplication complex, *cdc-6*, prevented the anchor cell from invading into the basement membrane in 32% of worms. The prereplication complex is a collection of proteins that bind to chromatin to initiate DNA replication and includes the origin recognition complex (ORC) proteins, *cdc-6* and *cdt-1*, and a hexamer of minichromosome maintenance (MCM) proteins. Knockdown of any of multiple components of the prereplication complex including Orc genes, *cdt-1* or *mcm-7*, resulted in a significant fraction of worms in which the anchor cell did not invade.

Further studies revealed that *mcm-7* plays a cell-autonomous role in anchor cell invasion unrelated to its role in DNA replication. *mcm-7* knockdown did not affect the worm’s ability to create an anchor cell or its cell cycle phases, but did prevent anchor cell polarization, formation of an actin-rich invasive protrusion, and expression of matrix metalloproteinases that help the anchor cell to infiltrate.

Protein components of the prereplication complex were previously shown to have functions in addition to their roles in the prereplication complex. In human cells, ORC6 knockdown resulted in not only decreased DNA replication, but also aberrant mitosis and multinucleated cells [5]. In *Drosophila*, ORC6 binds to a septin family protein and helps to ensure proper cell division [6]. Orc2 can bind to centromeres in S, G2, and M phases, and Orc2 inactivation resulted in failures of chromosome condensation and multiple centrosomes. High levels of MCM2, MCM3, and MCM7 were observed in medulloblastoma cancer cells, and high MCM3 expression was associated with poor prognosis [7]. In this study, overexpression of MCM2, MCM3, or MCM7 increased, and knockdown of these same genes decreased, the ability of medulloblastoma cells to migrate and invade.

One intriguing question is whether the prereplication complex proteins affect invasion as part of a protein complex, and if so, whether the invasion complex has the same protein composition and structure as the prereplication complex. While knockdown of multiple ORC genes, *cdc-6*, *cdt-1*, and *mcm-7*, all resulted in anchor cell invasion defects, knockdown of *mcm*’s other than *mcm-7* did not, suggesting an invasion protein complex might have a different structure from the prereplication complex. Consistent with this finding, chromosome abnormalities resulting in elevated levels of MCM7 were observed in medulloblastoma clinical samples, but no consistent changes affecting protein expression were observed for other MCM proteins [7]. Also of importance is the discovery by Lattmann and colleagues that reintroducing a mutated version of the MCM-7 protein unable to perform helicase function was not able to rescue the anchor cell invasion defect. This suggests that the catalytic activity of MCM-7 may be important for the invasion phenotype.

How do these prereplication complex proteins affect invasion in the anchor cell? One somewhat surprising and counterintuitive finding was that MCM-7 levels are not particularly high in anchor cells, suggesting that their levels may not be part of the invasive mechanism. Knockdown of MCM7 did result in a loss of the asymmetrical orientation of the catalytic and regulatory components of the PI3K complex. Hyperactivation of the PI3K pathway partially rescued the defects in invasion observed in the MCM-7 knockdown worms.

There are many more outstanding questions raised by these studies. How do MCM proteins regulate the asymmetrical organization of PI3K components, and is this the basis for their role in anchor cell invasion? Does a prereplication complex of proteins promote basement membrane invasion by other cells during development in *C*. *elegans* or other species? For instance, can prereplication complex proteins promote cancer cell metastasis?

Further, does using the same proteins, and possibly the same protein complex, for both DNA replication and invasion allow the cell to coordinate invasion with the cell cycle? Does this protect the cell from attempting to breach the basement membrane during DNA replication? Would invasion during DNA replication be deleterious for the cell? Do the many proteins with moonlighting functions associated with metabolism, which has also been reported to cycle [8], coordinate other cellular functions with the metabolic cycle? Finally, is the dual use of proteins with moonlighting functions a general property that allows cells to ensure that conflicting tasks are temporally dissociated?

## Abbreviations

MCM: minichromosome maintenance
ORC: origin recognition complex
TCA: tricarboxylic acid
